# Asymmetries in Global Perception Are Represented in Near- versus Far-Preferring Clusters in Human Visual Cortex

**DOI:** 10.1523/JNEUROSCI.2124-19.2019

**Published:** 2020-01-08

**Authors:** Shahin Nasr, Roger B.H. Tootell

**Affiliations:** Athinoula A. Martinos Center for Biomedical Imaging, Massachusetts General Hospital, Charlestown, Massachusetts 02129, and Department of Radiology, Harvard Medical School, Boston, Massachusetts 02115

**Keywords:** cortical columns, high-resolution fMRI, spatial frequency encoding, statistics of natural scenes, stereoselectivity

## Abstract

Human perception is more “global” when stimuli are viewed within the lower (rather than the upper) visual field. This phenomenon is typically considered as a 2-D phenomenon, likely due to differential neural processing within dorsal versus ventral cortical areas that represent lower versus upper visual fields, respectively. Here we test a novel hypothesis that this vertical asymmetry in global processing is a 3-D phenomenon associated with (1) higher ecological relevance of low-spatial frequency (SF) components in encoding near (compared with far) visual objects and (2) the fact that near objects are more frequently found in lower rather than upper visual fields. Using high-resolution fMRI, collected within an ultra-high-field (7 T) scanner, we found that the extent of vertical asymmetry in global visual processing in human subjects (*n* = 10) was correlated with the fMRI response evoked by disparity-varying stimuli in human cortical area V3A. We also found that near-preferring clusters in V3A, located within stereoselective cortical columns, responded more selectively than far-preferring clusters, to low-SF features. These findings support the hypothesis that vertical asymmetry in global processing is a 3-D (not a 2-D) phenomenon, associated with the function of the stereoselective columns within visual cortex, especially those located within visual area V3A.

**SIGNIFICANCE STATEMENT** Here we test and confirm a new hypothesis: fine-scale neural mechanisms underlying the vertical asymmetry in global visual processing. According to this hypothesis, the asymmetry in global visual processing is a 3-D (rather than a 2-D) phenomenon, reflected in the function of fine-scale cortical structures (clusters and columns) underlying depth perception. Our findings highlight the importance of considering these structures, as regions of interest, in clarifying the neural mechanisms underlying visual perception. The results also highlight the importance of statistics of natural scenes in shaping human visual perception.

## Introduction

Humans perceive visual stimuli more “globally” (as opposed to “locally”) when stimuli are presented within the lower visual field (LVF) compared with the upper visual field (UVF; Previc, 1990; Christman, 1993; Levine and McAnany, 2005). This vertical asymmetry is (at least partly) due to relatively higher sensitivity to lower spatial frequency (SF) components [≤1.0 cycles per degree (c/deg)], which are important for global processing (Shulman et al., 1986; Shulman and Wilson, 1987; LaGasse, 1993; Robertson et al., 1993; Flevaris et al., 2010), in LVF compared with UVF (Skrandies, 1987; Niebauer and Christman, 1998; Thomas and Elias, 2011). Although this asymmetry has been known for decades, its underlying neural mechanisms remain little known (Liu et al., 2006), partly due to technical challenges identifying the small (and spatially restricted) low- and higher-SF preferring sites within visual cortex (Tootell and Nasr, 2017). Such fine-scale structures may not be directly resolved using conventional neuroimaging techniques in humans.

Recently, by taking advantage of high-resolution fMRI, we showed that low-SF (≤0.3 c/deg) and higher SF-preferring clusters in humans are localized within thick- and thin-type columns (respectively) across areas V2, V3, and V3A (Tootell and Nasr, 2017). These thin- and thick-type columns were localized based on their stereoselectivity and color selectivity, respectively. Their columnar (radial) organization was shown based on (1) similarity between the activity patterns evoked across cortical layers and (2) comparison of the level of correlation across versus within layers (Nasr et al., 2016; Nasr and Tootell, 2018). The existence of these columns and their differential sensitivity to additional functional properties are consistent with findings based on invasive techniques in nonhuman primates (NHPs; for review, see Tootell and Nasr, 2017).

Here, we hypothesized that vertically asymmetric global processing is reflected in the activity evoked within stereoselective columns that represent LVF versus UVF across visual cortical areas. Since V3A showed a stronger low-SF selectivity compared with V2 and V3 (Gaska et al., 1988; Tootell and Nasr, 2017), we also expected a stronger link between the level of vertical asymmetry in global processing (i.e., behavior) and neural processing within V3A, compared with that in earlier visual areas.

An extension of this hypothesis is that this vertical asymmetry may be a 3-D (not 2-D) phenomenon, reflecting a hypothetically higher ecological relevance of low SFs in visual encoding within near, compared with far, space. This hypothesis is supported by several considerations. First, in real-life vision, near objects are more frequently located within the LVF compared with the UVF (Yang and Purves, 2003). Second, the perception of near (compared with far) objects may rely more on low-SF features likely due to spatial blurring—visual features emphasizing luminance-graded (e.g., low-SF) borders are generally perceived as nearer compared with those in which higher-SF features are relatively enhanced (Marshall et al., 1996; Mather and Smith, 2000; Sprague et al., 2016).

Our experiments specifically tested whether the asymmetry in global visual processing is reflected as a differential sensitivity to low-SF stimuli evoked within near- versus far-preferring clusters, distributed within stereoselective columns. Although NHP (Adams and Zeki, 2001; Tanabe et al., 2005; Chen et al., 2008) and human (Nasr and Tootell, 2018) studies reported that near- compared with far-preferring neurons are more frequently found in the cortical representation of the LVF compared with the UVF, to our knowledge, no previous study has compared the sensitivity of near- versus far-preferring neural clusters, in any visual area, at low SFs.

We scanned 10 human subjects using high-resolution fMRI. The results confirmed an association between vertical asymmetry in global processing and the response evoked by depth-varying stimuli within human V3A—a cortical area heavily involved in stereovision (Tsao et al., 2003; Neri et al., 2004; Goncalves et al., 2015; Tootell and Nasr, 2017). In subsequent experiments, we showed a higher sensitivity to low-SF features in near- compared with far-preferring clusters. These findings suggest that the vertical asymmetry in global visual processing is a 3-D phenomenon, associated with differential low-SF sensitivity of near- versus far-preferring clusters.

## Materials and Methods

### 

#### Participants

Eighteen human subjects (9 female), 22–40 years of age, participated in the behavioral test (Experiment 1). Ten of 19 subjects (5 female) were selected (regardless of their behavioral performance) to participate in the subsequent imaging test (Experiment 2). Among them, eight subjects (four female), also agreed to participate in Experiments 3 and 4. All subjects had normal or corrected-to-normal vision and radiologically normal brains, without any history of neuropsychological disorder. All experimental procedures conformed to NIH guidelines and were approved by Massachusetts General Hospital protocols. Written informed consent was obtained from all subjects before the experiments.

#### General procedures

Each subject was scanned multiple times (on different days) in a Siemens 7 T scanner (Siemens Healthcare) to localize the stereoselective thick-type columns (Nasr et al., 2016) and near- and far-preferring clusters within them (two sessions), and also to localize color-selective thin-type columns (one session; Nasr et al., 2016). Eight of 10 subjects also participated in additional scan sessions to measure sensitivity to 2-D spatial frequency (two sessions) and 3-D spatial configuration (one session) within their near- and far-preferring clusters. All 10 subjects were also scanned in a 3 T scanner (TIM Trio, Siemens Healthcare) in one additional session, for structural and retinotopic mapping (one session).

#### Visual stimuli

During the behavioral test outside the scanner, stimuli were presented via an LCD monitor, positioned 57 cm in front of the viewers. Subjects were instructed to position their chins on a chin rest to fix their head position relative to the monitor.

Inside the scanner, stimuli were presented via a projector (1024 × 768 pixel resolution, 60 Hz refresh rate) onto a rear-projection screen, viewed through a mirror mounted on the receive coil array. In MATLAB 2018a (MathWorks), The Psychophysics Toolbox (Brainard, 1997; Pelli, 1997) was used to control stimulus presentation.

##### Experiment 1.

**Figure 1.**
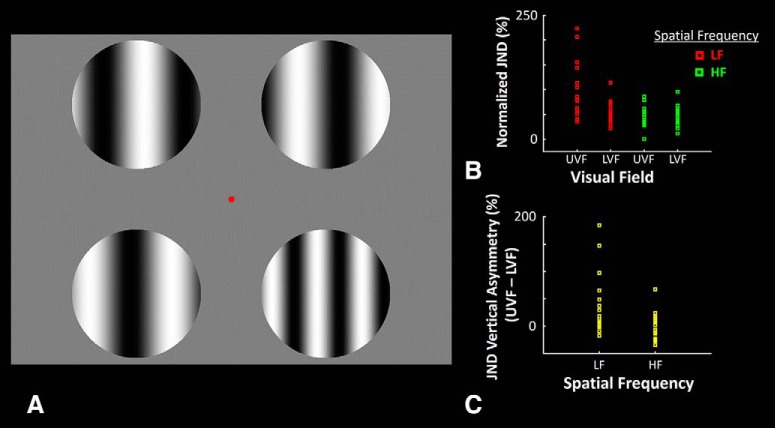
A schematic representation of the behavioral paradigm used in Experiment 1, along with subjects' behavioral performance during those tests. ***A***, The stimuli are presented in one exemplar trial, during which subjects were instructed to look at the fixation spot (small red dot) presented at the center of screen and detect the hemifield (UVF vs LVF) in which the “odd” grating was presented. ***B***, The individually normalized JND for discriminating low- and high-SF gratings in LVF and UVF. On average, subjects showed a higher JND for discriminating low-SF (but not high-SF) gratings in UVF compared with LVF (i.e., a vertical symmetry). ***C***, This asymmetry is more directly shown by presenting the measured difference in JND between UVF and LVF for discriminating the low- and high-SF gratings. Error bars represent 1 SEM.

Outside the scanner, we measured each subjects' threshold as a “just noticeable difference” (JND) in detecting a small change in SF in the upper versus lower hemifields. In each trial, subjects were presented simultaneously with four gratings of either low (0.3 c/deg) or higher (3.0 c/deg) SF with different phase values, plus a small fixation target (0.1° × 0.1°) presented at the center of screen (Fig. 1). Each grating was presented within a circular aperture (*r* = 3.9°), positioned in one corner of screen, with a center-to-center distance of 11.4° between adjacent apertures. Gratings were presented only for a short time (100 ms), remained without any change (i.e., statically) within the trial, then were followed by presentation of a spatially uniform gray screen.

Before the onset of each trial, one of the four target locations was chosen randomly (25% chance), and the SF of the grating in that location was changed (i.e., increased or decreased) slightly relative to the SF in the other three apertures. The level of this change (ΔSF) was controlled using a staircase method (Pelli, 1997) to adjust each subject's response accuracy toward 75%. This adjustment was made between trials, independently for low versus higher SFs and for LVF versus UVF (i.e., 2 × 2 design). Subjects were instructed to report the hemifield (upper vs lower) in which the SF of the grating differed from the others by pressing a key on the keypad, in a two-alternative forced choice (2AFC) procedure. The subsequent trial began with a delay (1.5 ± 0.5 s) following a subject's response. This procedure increased the overall efficiency (compared with testing left and right hemifields separately). Moreover, use of the spatially balanced design, plus the short stimulus presentation time (100 ms), likely helps to control eye movement stability, compared with testing stimulus locations in isolation. Each subject participated in 50 trials per condition (i.e., 200 trials in total). Accuracy was stressed more than speed.

##### Experiment 2.

In different blocks, subjects were presented with random dot stereogram (RDS; Julesz, 1971) stimuli, extending 20° × 20° in the visual field. These stimuli formed a stereoscopic percept of a regular array of depth-based cuboids that varied sinusoidally either in front (near; 0°–0.22°) or behind (far; −0.22°–0°) the fixation spot with independent phase, similar to stimuli described earlier (Tsao et al., 2003; Neri et al., 2004; Bridge and Parker, 2007; Minini et al., 2010; Nasr and Tootell, 2018). In the control condition, the fused percept formed a 2-D frontoparallel plane intersecting the fixation target (i.e., zero depth at that point). Subjects viewed the stimuli through custom anaglyph spectacles using Kodak Wratten filters No. 25 (red) and No. 44A (cyan). Each run consisted of nine blocks of 24 s each, showing one of the three possible stimulus conditions (i.e., three repetitions for each condition). The sequence of blocks was randomized. Each run began and ended with an additional block (12 s) showing a spatially uniform (“blank”) achromatic field. Each subject participated in 12 runs per session, during which 960 functional volumes were collected (for more details, see Nasr and Tootell, 2018).

##### Experiment 3.

In a block design experiment, subjects were presented with gratings of differing SFs (0.1, 0.27, 0.73, 2.08, and 5.79 c/deg) and achromatic contrasts (1.43%, 5.25%, 15.95%, 50.14%, and 99. 62%) across different blocks, in a 5 × 5 design (i.e., 25 different experimental conditions), extending 20° × 20° in the visual field. In each block, grating orientation and phase changed every 4 and 1 s, respectively. Each run consisted of 15 blocks of 16 s each, showing 1 of the 25 possible stimulus conditions without any repetition within the run. Each run began and ended with an additional block (12 s) of uniform gray of equivalent mean luminance. During these runs, subjects were required to do a color change detection for a very small, centrally located fixation spot whose color varied between dark versus light green every few seconds (1.5 ± 0.5 s). Each subject participated in two scan sessions, with 12 runs (1056 functional volumes) per session.

##### Experiment 4.

Stimuli were 3-D checkerboards, generated based on sparse (5%) RDS. Across different blocks, check size was varied, forming different 3-D cubic arrangements (3 × 4, 6 × 8, 12 × 16, and 24 × 32). Within each block, the level of disparity varied between 0.22° and −0.22° and stimuli extended 20° × 20° in the visual field. In the control condition, presented within separate blocks, the RDS stimuli formed a uniform (1 × 1) 2-D frontoparallel plane intersecting the fixation target (i.e., zero depth at that point; i.e., five experimental conditions in total). Each experimental run included 10 blocks (24 s/block), plus 12 s of uniform blank presentation at the beginning and end of the runs. Each subject participated in 12 runs per session, during which 1248 functional volumes were collected. During these runs, subjects were required to do a shape change detection task for a centrally selected fixation spot, whose shape varied between a circle and a square every seconds few (1.5 ± 0.5 s).

##### Localizer scans.

To localize stereoselective columns, subjects were presented with RDS stimuli, extending 20° × 20° in the visual field. In 50% of blocks, these stimuli formed a stereoscopic percept of a regular array of depth-based cuboids that varied sinusoidally (between 0.22° and 0.22°) relative to the frontoparallel plane that intersected the fixation spot, similar to a stimulus described earlier (Tsao et al., 2003; Neri et al., 2004; Bridge and Parker, 2007; Minini et al., 2010). Importantly, these stimuli differed from those used in Experiment 2, in which the disparity level ranged between either −0.22° to 0° or 0° to −0.22° across different blocks. Data from Experiments 2 and 4 were collected during independent scan sessions.

In the other half of each block, the fused percept formed a 2-D frontoparallel plane intersecting the fixation target (i.e., zero depth at that point). Subjects viewed the stimuli through custom anaglyph spectacles using Kodak Wratten filters No. 25 (red) and No. 44A (cyan). Other details, such as the stimuli and the paradigm, have been reported previously (Nasr et al., 2016). The results of Experiment 2 were used to detect and localize near- versus far-preferring clusters within the stereoselective (thick-type) columns (Nasr et al., 2016; Nasr and Tootell, 2018).

To ensure that these stereoselective columns did not overlap with the color-selective columns, we also localized color-selective (thin-type) columns. Briefly, in different blocks, subjects were presented with isoluminance color-varying and luminance-varying gratings. All stimuli extended 20° × 20° in the visual field. Grating stimuli were presented at different orientations (0°, 45°, 90°, or 135°), drifting in orthogonal directions (reversed every 6 s) at 4 deg/s. Vertices that showed overlapping stereoselective and color-selective activity were excluded from our analysis. Other details have been reported previously (Nasr et al., 2016).

##### Retinotopic mapping.

Details of retinotopic mapping have also been reported previously (Nasr et al., 2011). Briefly, stimuli were color- and luminance-varying images of scenes and face mosaics, which were presented within retinotopically limited apertures, against a gray background. The retinotopic apertures included horizontal/vertical meridian (radius, 10°; polar angle, 30°) wedges, upper/lower hemifield (radius, 10°; polar angle, 150°) wedges, a foveal disk (radius, 0°–1.5°), and a peripheral ring (radius, 5°–10°), presented across different blocks. To confirm the borders of retinotopic areas, in two subjects we also presented phase-encoded, contrast-reversing (1 Hz) checkerboards within continuously rotating rays or continuously expanding/contracting ring stimuli, in different runs/experiments. Details of this procedure have been described previously (Sereno et al., 1995).

#### Imaging

The main MRI experiments were conducted in a 7 T Siemens whole-body scanner equipped with SC72 body gradients (70 mT/m maximum gradient strength and 200 T/m/s maximum slew rate) using a custom-built 32-channel helmet receive coil array, and a birdcage volume transmit coil (Keil et al., 2010). To acquire functional images, we used a single-shot gradient-echo EPI with 1.0 mm isotropic voxels with the following protocol parameter values: TR = 3000 ms; TE = 28 ms; flip angle = 78°; matrix = 192 × 192; band width (BW) = 1184 Hz/pixel, echo-spacing = 1 ms; 7/8 phase partial Fourier; FOV = 192 × 192 mm; 44 oblique-coronal slices; and acceleration factor *r* = 4 with GRAPPA (generalized autocalibrating partially parallel acquisitions) reconstruction and FLEET (fast low-angle excitation echoplanar technique)-ACS (autocalibration signal) data (Polimeni et al., 2016) with a 10° flip angle. The field of view fully covered the occipital cortical areas V1, V2, V3, and V3A (but not always MT).

Retinotopic mapping was conducted using a 3 T Siemens scanner (TIM Trio) and the vendor-supplied 32-channel receive coil array. Functional data were acquired using single-shot gradient-echo EPI, with nominally 3.0 mm isotropic voxels using the following protocol parameters: TR = 2000 ms; TE = 30 ms; flip angle = 90°; matrix = 64 × 64; BW = 2298 Hz/pixel; echo-spacing = 0.5 ms, without partial Fourier, FOV = 192 × 192 mm; 33 axial slices covering the entire brain; and no acceleration.

Structural (anatomical) data were acquired using a 3-D T1-weighted MPRAGE sequence with the following protocol parameter values: TR = 2530 ms; TE = 3.39 ms; TI = 1100 ms; flip angle = 7°; BW = 200 Hz/pixel, echo spacing = 8.2 ms; voxel size = 1.0 × 1.0 × 1.33 mm^3^; and FOV = 256 × 256 × 170 mm^3^.

#### Data analysis

Functional and anatomical MRI data were preprocessed and analyzed using FreeSurfer and FS-FAST (version 6.0; http://surfer.nmr.mgh.harvard.edu/; Fischl, 2012). For each subject, inflated and flattened cortical surfaces were reconstructed based on the high-resolution anatomical data (Dale et al., 1999; Fischl et al., 1999, 2002).

All functional images were corrected for motion artifacts. The 3 T functional data, collected for retinotopic mapping, were spatially smoothed (Gaussian filtered with a 5 mm FWHM). However, no spatial smoothing was applied to the main imaging data acquired at 7 T (i.e., 0 mm FWHM). For each subject, functional data from each run were rigidly aligned (6 df) relative to his/her own structural scan, using rigid boundary-based registration (Greve and Fischl, 2009). This procedure enabled us to combine and average data collected for each subject across multiple scan sessions.

A standard hemodynamic model based on a gamma function was fit to the fMRI signal to estimate the amplitude of the BOLD response (Boynton et al., 1996; Courtney et al., 1997; Dale and Buckner, 1997). For each individual subject, the average BOLD response maps were calculated for each condition (Friston et al., 1999). Finally, voxelwise statistical tests were conducted by computing contrasts based on a univariate general linear model, and the resultant significance maps were projected onto the subject's anatomical volumes and reconstructed cortical surfaces.

To reduce the impact of pial veins on evoked activity maps (Yacoub et al., 2008; Polimeni et al., 2010; Nasr et al., 2016), brain activity was sampled from the deepest (as opposed to more superficial) cortical layers. Accordingly, for each subject the gray matter–white matter (“deep”) interface was detected based on their own high-resolution structural scans (see above) using FreeSurfer (Dale et al., 1999; Fischl et al., 1999, 2002). To measure the fMRI activity, the percentage fMRI signal change was calculated for those functional voxels that intersected this gray matter–white matter interface, and the resultant values were projected onto the corresponding vertices of the surface mesh.

#### Region of interest

Borders of regions of interest (ROIs), including V1, V2, V3, and V3A, were defined for each subject based on her/his own retinotopic mapping (see above). Stereoselective columns were localized functionally by contrasting the activity evoked by disparity-varying RDS that formed 3-D versus 2-D stimuli (Nasr et al., 2016). Near- versus far-preferring clusters within the stereoselective columns were also localized functionally during a separate set of scans by contrasting the activity evoked by disparity-varying RDSs that formed near versus far stimuli (Experiment 2; Nasr and Tootell, 2018).

#### Statistical analysis

Statistical tests were based on repeated-measures ANOVA. When necessary (based on the Mauchly's test), results were corrected for violation of the sphericity assumption, using the Greenhouse–Geisser method. In Experiment 3, to test the reliability of potential effects, a group factor (first vs second scan) was also considered in all ANOVA tests. In an ROI-based analysis, we did not find any difference between activity evoked within left versus right hemispheres. Therefore, data from both hemispheres were averaged together to enhance the signal-to-noise ratio. In all cases, data were tested for normality before choosing the statistical test (*p* < 0.05 was considered significant).

To test the association between the evoked fMRI activity and the subject's behavior (Experiment 2), we used a linear regression model (behavior ∼1 + fMRI + fMRI × cluster type). This analysis enabled us to test whether behavior showed a different type of association with activity evoked within each cluster type (near- vs far-preferring). All resultant *p* values were corrected for multiple comparisons (V2 vs V3 vs V3A), using a Bonferroni correction method. Subsequently, to test the significance of differences between the correlations across visual areas (see Results), we used a second linear regression model (behavior ∼1 + fMRI + fMRI × cluster type + fMRI × area + fMRI × cluster type × area). All statistical analyses were conducted using the MATLAB (2018a) Statistics and Machine Learning Toolbox (MathWorks).

#### Data availability

Data are available on request from the authors.

## Results

### Experiment 1: vertical asymmetry for perception of low SF

We conducted psychophysical measurements in 19 participants to measure the level of vertical asymmetry in global perception, using stimuli matching those used later during fMRI experiments. A 2AFC procedure was used to measure the JND in discriminating variations in SF, centered at either low (0.3 c/deg) or higher (3.0 c/deg) SFs (i.e., 100 × ΔSF/SF), at 75% response accuracy (see Materials and Methods). Consistent with previous studies, subjects showed a higher JND in the UVF compared with the LVF for discriminating low SFs, but not higher SF gratings (Fig. 1). We applied a two-way repeated-measures ANOVA [hemifield (LVF vs UVF) and SF (low vs higher)] to the measured thresholds, which showed significant effects of hemifield (*F*_(1,18)_ = 5.26, *p* = 0.03), SF (*F*_(1,18)_ = 16.22, *p* < 10^−3^), and SF × hemifield (*F*_(1,18)_ = 4.47, *p* = 0.04). These results confirmed that this vertical asymmetry was selective for low-SF stimuli, rather than a broader response bias.

### Experiment 2: is the asymmetry in low-SF processing associated with activity within near- versus far-preferring clusters?

**Figure 2.**
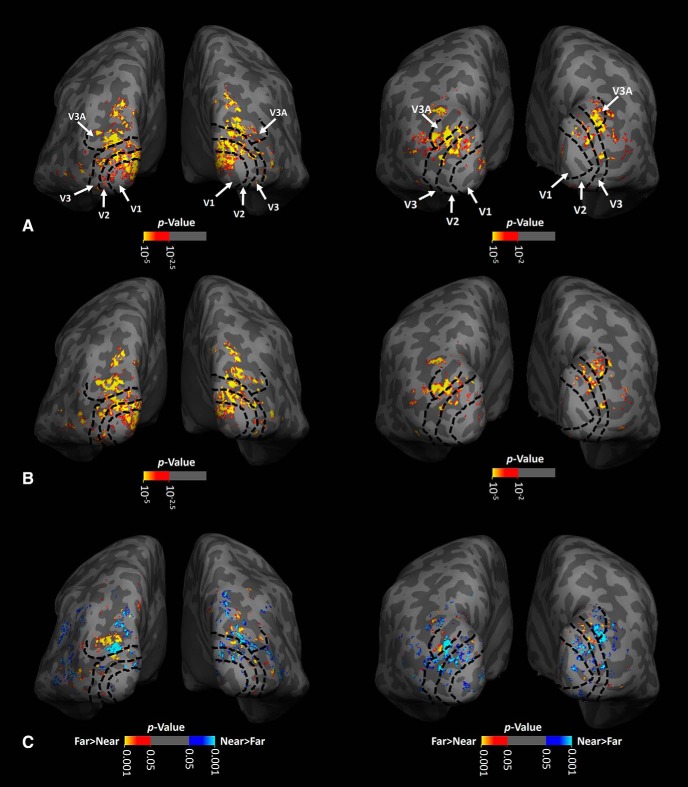
Activity evoked by depth-varying RDS stimuli within the occipital visual cortex of two individual subjects (shown on left and right, respectively), each overlaid on their own inflated brains. ***A***, ***B***, Activity evoked by disparity-varying stimuli ranging between 0° and 0.4° (i.e., near space) and −0.4° to 0° (i.e., far space), respectively, all compared with activity evoked by an otherwise equivalent stimuli presented at a constant frontoparallel plane that intersected the fixation spot (i.e., zero depth). Consistent with previous studies in humans at both high (Nasr et al., 2016; Tootell and Nasr, 2017; Nasr and Tootell, 2018) and conventional (Tsao et al., 2003; Neri et al., 2004) fMRI resolution, we found depth-sensitive activity within visual areas V1, V2, V3, and V3A (see Materials and Methods). ***C***, Activity evoked by the more detailed contrast of “far–near”-selective activity, within occipital visual cortex of the same individuals. Near-preferring (blue to cyan) and far-preferring (red to yellow) clusters can also be found in different visual areas. For each subject, the borders of retinotopic visual areas (black dashed lines) are defined based on an independently collected set of retinotopic stimuli and scans.

**Figure 3.**
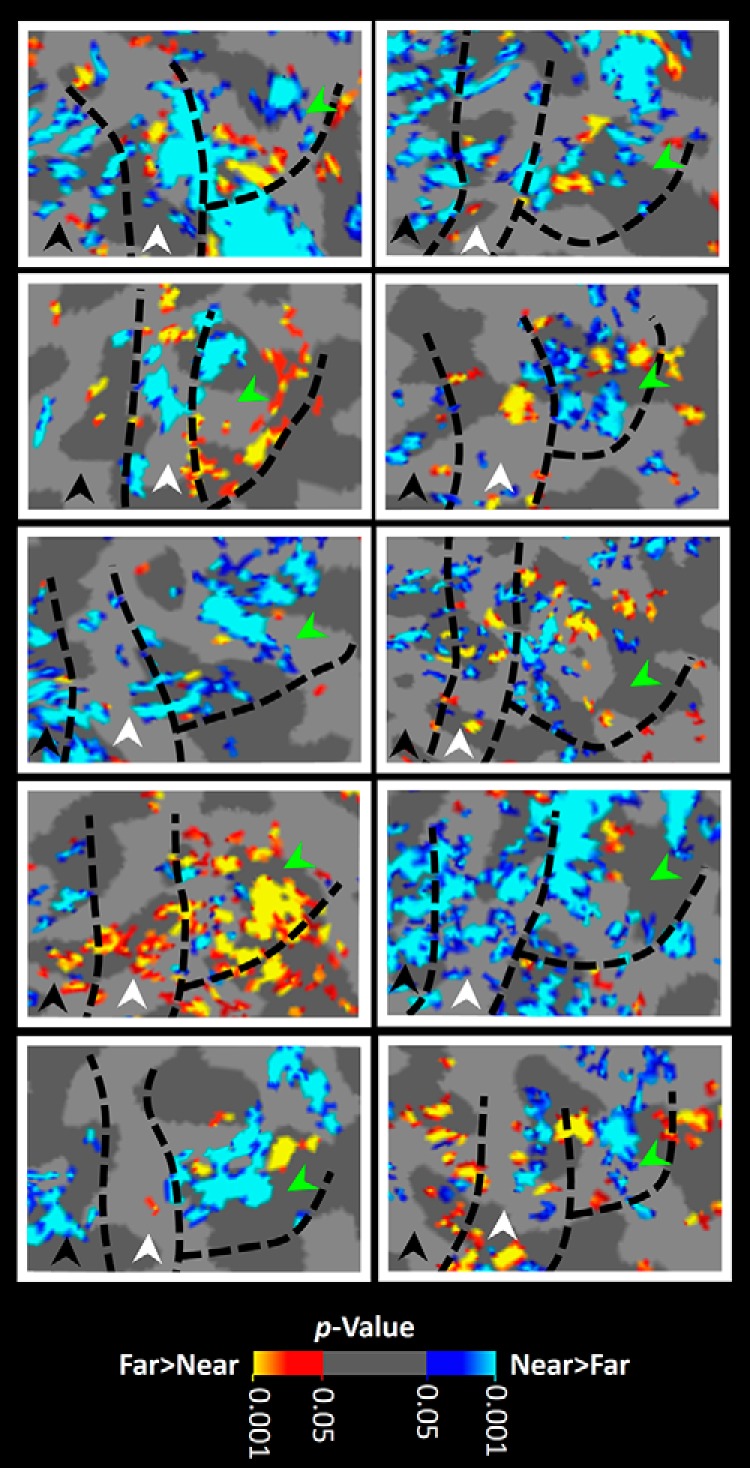
Distribution of near- and far-preferring clusters within areas V2, V3, and V3A. Each panel shows activity evoked by the far–near contrast in one hemisphere, overlaid on each subject's own “flattened” cortex, rather than the “inflated” cortical surface shown in Figure 2. In V3A, near-preferring activity (blue to cyan) occupied a larger portion of the surface area (here, and across a range of thresholds) compared with far-preferring (red to yellow) clusters, on average. The location of near- and far-preferring clusters across individuals showed no systematic organization, at least in the current sample. In each panel, areas V2, V3, and V3A are indicated by black, white, and green arrowheads, respectively. Other details are similar to those in Figure 2.

**Figure 4.**
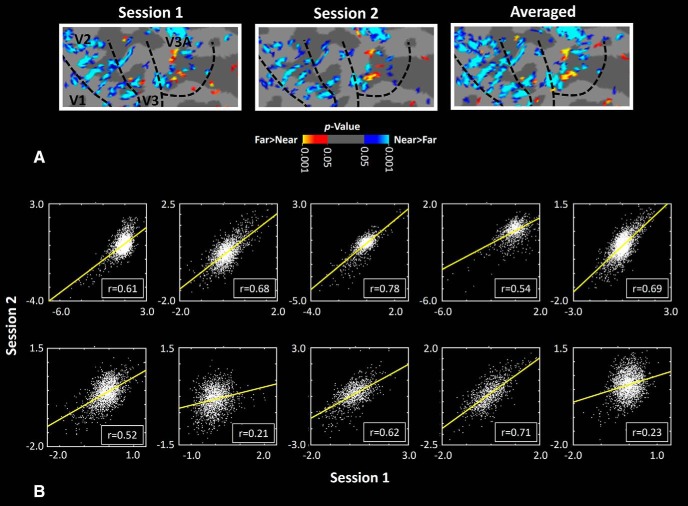
Reproducibility of a near- versus far-preferring clusters map in V3A across different subjects. ***A***, Similarity between the activity maps evoked by far–near contrast in one individual across the two sessions. ***B***, Correlation between the percentage of fMRI signal change in each V3A vertex, evoked by near versus far contrast during the first versus the second scan sessions in each individual subject. A Pearson test of correlation in data collected from each subject showed a significant (*r* > 0.23; *p* < 10^−4^) correlation between activity evoked across these two sessions. Since it could be argued that the potential correlation within/between sessions was complicated by nonindependence of activity in adjacent vertices, we also used a stricter test in which (instead of using all V3A vertices), we randomly selected 10% of vertices, and measured the level of correlation between their activity across the two sessions. These correlation coefficients were compared relative to the chance level, defined as the level of correlation after randomly misaligning (i.e., spatially “shuffling”) V3A vertices between the two sessions. We repeated this test 10,000 times for each subject. In all subjects, we found the probability of finding a correlation coefficient that was less than the correlation coefficient of misaligned vertices (i.e., the null hypothesis) was <0.01. These results support the reliability/reproducibility of these activity maps between scan sessions. Data from both hemispheres are combined in these analyses.

Next, we tested whether the level of asymmetric low-SF sensitivity (measured behaviorally) was associated with the fMRI response evoked within stereoselective columns (*n* = 10). In other words, we tested whether subjects who show a stronger stereoselective activity also show a stronger asymmetric low-SF sensitivity. Figures 2 and 3 show activity evoked by depth-varying stimuli (see Materials and Methods) within near versus far distances across V1, V2, V3, and V3A. These maps of near versus far activity were robust and consistent across the two scan sessions (see Materials and Methods) in all individuals (Fig. 4). A similar consistency (across sessions) was noted in maps in V2 and V3 (Nasr et al., 2016).

**Figure 5.**
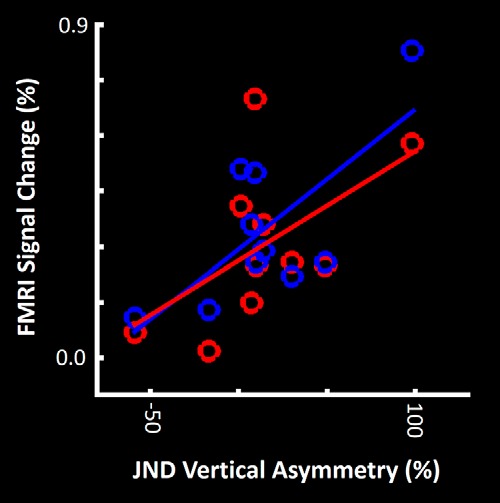
The amount of vertical asymmetry in global visual processing is associated with the level of depth-sensitive fMRI activity evoked within area V3A. Subjects that showed a stronger stereoselective fMRI activity (evoked by depth-varying RDS stimuli) showed a stronger difference between JNDs for low-SF discrimination (an index of global visual processing) in LVF compared with UVF (Fig. 1). Red and blue circles show selective activity evoked in one subject by near and far stimuli, respectively. All fMRI values were measured relative to the activity evoked by zero-depth RDS stimuli.

Consistent with our hypothesis, the association between vertical asymmetry in global processing and the function of the stereoselective cortical columns, we found a significant correlation between the level of vertical asymmetry in low-SF discrimination [measured behaviorally as UVF_JND_-LVF_JND_ (Experiment 1)] and the level of stereoselective fMRI activity evoked within V3A. Linear regression showed a significantly higher vertical asymmetry in low-SF discrimination in those subjects who showed a stronger stereoselective response (β = 0.64; *p* < 0.01; corrected for multiple comparison; see below). This association was similar for activity evoked by “near-zero disparity” and “far-zero disparity” contrasts, without any significant moderator effect of depth direction (i.e., near vs far, *p* = 0.73; Fig. 5).

Next, we tested whether this correlation was quantitatively limited to cortical area V3A, or whether it was evident in lower-tier cortical areas V2 and V3. In both of those areas, the analysis used above did not yield any significant correlation between the subject's vertical asymmetry and the stereoselective fMRI response evoked within stereoselective (thick-type) columns within V2 (*p* = 0.12) or V3 (*p* = 0.15). All *p* values were corrected for multiple comparisons using a Bonferroni correction.

Then, to test whether this difference between correlation values across areas V2 versus V3 versus V3A was significant, we used a second regression model in which each visual area was considered as an independent factor (see Materials and Methods). The results showed a significant moderator effect of visual area (i.e., behavior ∼1 + fMRI × area; β = 0.45; *p* < 10^−3^). Thus, vertical asymmetry in the low-SF JNDs was more directly associated with activity evoked within V3A, compared with earlier visual areas V2 and V3.

### Experiment 3: SF preference in near- versus far-preferring clusters based on 2-D stimuli

The results of Experiments 1 and 2 suggest that at least one aspect of vertical asymmetry in global processing (i.e., sensitivity to low-SFs) is predictable based on the fMRI response evoked within area V3A by depth-varying stimuli. Here, we tested for a difference between low-SF selectivity of near- versus far-preferring clusters, as suggested by our hypothesized 3-D mechanism for this vertical asymmetry. fMRI activity was measured within near- and far-preferring clusters in response to sinusoidal gratings with different SFs (0.10, 0.27, 0.73, 2.08, and 5.79 c/deg) and contrasts (1.40%, 5.25%, 16.00%, 50.10%, and 99.60%), presented in a 5 × 5 blocked design (see Materials and Methods). Analyses focused on V3A, but included V2 and V3 as well, partly as control conditions. To increase the level of signal averaging, and to measure the reliability of the results, each subject was scanned twice, on different days.

**Figure 6.**
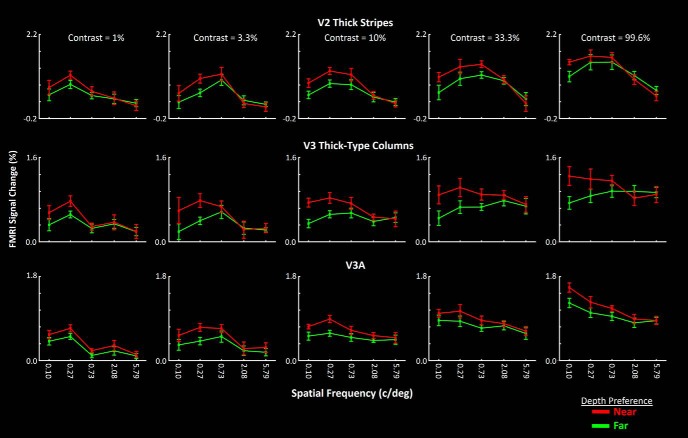
Near-preferring clusters show a higher low-SF selectivity compared with far-preferring clusters. Each panel shows the activity evoked by gratings of various SFs, at one contrast level. Stronger low-SF selectivity of near-preferring clusters can be seen in V2, V3, and V3A, across different stimulus contrast levels. Area V3A showed a preference for lower SFs, compared with V2 and V3. Activity within near- and far-preferring clusters is indicated by red and green lines, respectively. Error bars represent 1 SEM.

In all three cortical areas, the results showed that the higher sensitivity to low-SF components was more prominent in near-preferring compared with far-preferring clusters, especially at higher contrasts (Fig. 6). A three-factor repeated-measures ANOVA [SF, contrast and cluster type (near vs far preferring)], with a group factor (first vs second session), showed a significant effect of cluster type × SF interaction (*F*_(4,48)_ > 7.13, *p* < 10^−3^) on activity measured within V2, V3, and V3A. This result suggests a higher fMRI response to lower-SF stimuli in near-preferring compared with far-preferring clusters (Table 1). These results also support the hypothesis that the asymmetric visual global processing reflects a stronger low-SF sensitivity in near-preferring compared with far-preferring clusters. In neither ROI did we find a significant interaction between the effect of scan sequence and the other independent factors (*p* > 0.24), thus supporting the reliability of our findings.

**Table 1.** SF sensitivity across near- versus far-preferring clusters within areas V2, V3, and V3A (Experiment 3)

	V2		V3		V3A	
	*F* Value	*p* Value	*F* Value	*p* Value	*F* Value	*p* Value
Scan sequence (first vs second scan)	2.11	0.17	1.09	0.32	1.54	0.24
SF (0.10 vs 0.27 vs 0.73 vs 2.08 vs 5.79 c/deg)	40.39	<10^−7^	5.38	0.02	15.97	<10^−4^
SF × scan sequence	0.67	0.52	1.07	0.38	0.48	0.63
Contrast (1.43%, 5.25%, 15.95%, 50.14%, and 99. 62%)	19.32	<10^−4^	38.35	<10^−6^	128.27	<10^−12^
Contrast × scan sequence	0.25	0.73	0.21	0.93	0.36	0.70
Cluster type (near vs far-preferring stereoselective)	39.72	<10^−4^	5.21	0.04	9.29	0.01
Cluster type × scan sequence	1.73	0.21	0.34	0.57	0.38	0.55
SF × contrast	1.91	0.09	1.12	0.33	2.48	0.04
SF × contrast × scan sequence	1.03	0.42	0.94	0.47	1.24	0.30
SF × cluster type	16.36	<10^−3^	7.13	<10^−3^	20.13	<10^−6^
SF × cluster type × scan sequence	0.19	0.74	0.02	0.99	0.78	0.49
Contrast × cluster type	2.29	0.11	2.95	0.08	0.84	0.45
Contrast × cluster type × scan sequence	0.22	0.85	0.30	0.71	0.84	0.45
SF × contrast × cluster type	3.15	0.02	2.47	0.08	2.32	0.04
SF × contrast × cluster type × scan sequence	0.81	0.53	0.92	0.44	0.78	0.59

The results of this experiment also showed a systematic shift in the preferred SF, in V2 (0.27 c/deg), and V3 and V3A (0.10 c/deg), regardless of the visual depth preference of the ROI (Fig. 6). Consistent with this observation, a three-factors repeated-measures ANOVA (SF and cluster type and area) with a group factor (first vs second scan session), applied to activity evoked by high contrast (contrast = 99.6%), stimuli showed a significant interaction between SF × area effects (*F*_(8,96)_ = 18.55, *p* < 10^−5^; Table 2). Thus, a stronger correlation between the V3A fMRI response and asymmetric global processing (indexed by low-SF components) compared with lower stage visual areas could be due to a lower SF preference in V3A compared with earlier areas (see also Experiment 4). In all of these tests, the effect of group, and its interaction with other factors, remained nonsignificant (*p* > 0.27), thus supporting the reliability of our findings.

**Table 2.** SF sensitivity across near- versus far-preferring clusters within areas V2, V3, and V3A (Experiment 3)

	*F* Value	*p* Value
Scan sequence (first vs second scan)	1.31	0.27
Area (V2 vs V3 vs V3A)	0.39	0.67
Area × scan sequence	0.14	0.86
SF (0.10 vs 0.27 vs 0.73 vs 2.08 vs 5.79 c/deg)	9.99	<10^−3^
SF × scan sequence	0.60	0.58
Cluster type (near vs far-preferring stereoselective)	16.72	<0.01
Cluster type × scan sequence	0.03	0.87
Area × SF	18.55	<10^−5^
Area × SF × scan sequence	0.29	0.79
Area × cluster type	0.02	0.93
Area × cluster type × scan sequence	0.44	0.56
Cluster type × SF	10.64	<0.01
Cluster type × SF × scan sequence	0.20	0.72
Area × cluster type × SF	1.55	0.23
Area × cluster type × SF × scan sequence	0.21	0.82

In cortical areas V2 and V3, the retinotopic mapping of upper versus lower visual fields is more precise compared with higher-tier areas (Engel et al., 1997; Wade et al., 2002; Sereno et al., 2013). This is due to the increase in receptive field size and scatter in higher-level compared with lower-level visual areas (Hubel and Wiesel, 1974; Gattass et al., 1987; Smith et al., 2001; Dumoulin and Wandell, 2008) that affects the localization of dorsal versus ventral borders in higher-level visual areas including V3A. At least in V2 and V3, it could be argued that this differential low-SF sensitivity between near- and far-preferring clusters could reflect a heterogeneous distribution of near- and far-preferring clusters within the cortical representations of the LVF and UVF (Nasr and Tootell, 2018).

To address this possibility, we tested whether the observed differences between the low SF-related response in near- versus far-preferring clusters was due to a secondary difference between activity evoked within “dorsal versus ventral” stereoselective columns, independent of their “near versus far” depth preference. In this test, dorsal versus ventral stereoselective columns were individually defined for each subject, based on the location of stereoselective columns relative to the cortical representation of the lower versus upper visual field, mapped retinotopically (see Materials and Methods). The above analysis was applied to activity measured within dorsal versus ventral (rather than near vs far) stereoselective columns in V2 and V3. The results showed neither a significant cluster type × SF (*F*_(4,48)_ < 2.57, *p* > 0.12) nor cluster type × SF × contrast (*F*_(16,192)_ < 1.69, *p* > 0.15) interactions (Table 3).

**Table 3.** SF sensitivity across dorsal versus ventral stereoselective columns within areas V2 and V3 (Experiment 3)

	V2		V3	
	*F* Value	*p* Value	*F* Value	*p* Value
Scan sequence (first vs second scan)	3.23	0.10	2.71	0.13
SF (0.10 vs 0.27 vs 0.73 vs 2.08 vs 5.79 c/deg)	50.62	<10^−8^	14.06	<10^−3^
SF × scan sequence	0.54	0.59	1.11	0.34
Contrast (1.43, 5.25, 15.95, 50.14, and 99.62%)	26.39	<10^−5^	45.40	<10^−8^
Contrast × scan sequence (first vs second scan)	0.25	0.76	0.54	0.60
Cluster type (dorsal vs ventral stereoselective columns)	5.31	0.04	0.87	0.37
Cluster type × scan sequence	0.12	0.73	0.37	0.55
SF × Contrast	3.90	<0.01	1.36	0.24
SF × Contrast × scan sequence	0.91	0.48	0.77	0.59
SF × cluster type	0.48	0.61	2.57	0.12
SF × cluster type × scan sequence	0.24	0.78	0.08	0.85
Contrast × cluster type	4.33	0.02	3.40	0.04
Contrast × cluster type × scan sequence	0.09	0.92	0.82	0.45
SF × Contrast × cluster type	1.47	0.20	1.69	0.15
SF × Contrast × cluster type × scan sequence	0.72	0.63	0.70	0.61

**Figure 7.**
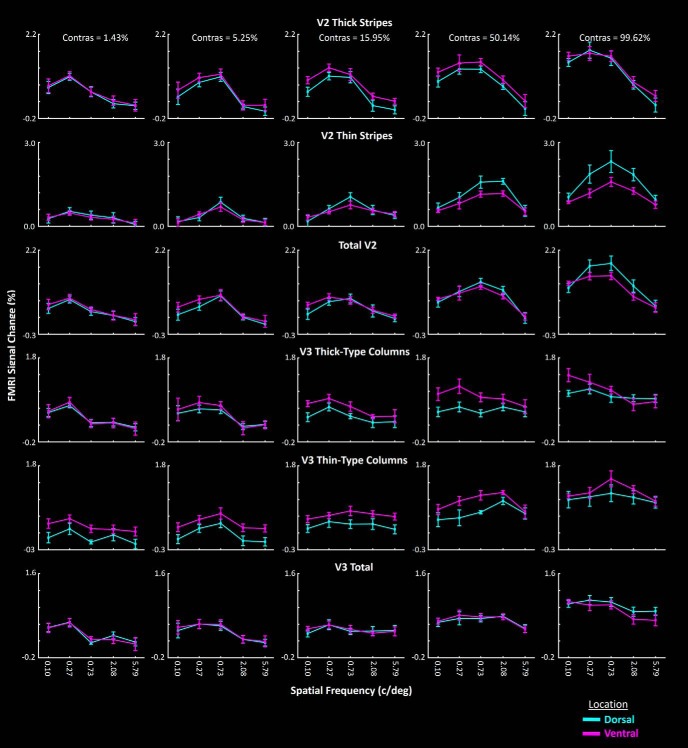
Response to gratings of various SFs within dorsal versus ventral regions (LVF vs UVF cortical representations, respectively) of areas V2 and V3. In contrast to near- and far-preferring clusters (Fig. 6), these areas did not show greater activity within dorsal compared with ventral depth-selective regions in response to lower-SF gratings. Similar results were also found when comparing the activity evoked within dorsal versus ventral portions of V2 and V3, regardless of their depth selectivity. These results ruled out the possibility that the asymmetry found in Experiment 3 (i.e., higher low-SF selectivity in near-preferring compared with far-preferring clusters) is a second-order phenomenon relative to the potential difference between activity evoked within dorsal versus ventral regions. Error bars represent 1 SEM.

We then applied the same test to activity measured in color-selective columns and/or the whole visual areas (see Materials and Methods). We found no apparent difference between the response to low-SF gratings in dorsal versus ventral areas (Fig. 7) in any of these areas. We found only a stronger sensitivity to mid-SF (0.73 c/deg) gratings in V2 dorsal versus ventral color-selective columns. However, V3 color-selective columns showed an opposite effect, making it unlikely that color-selective columns contribute to vertically asymmetric global configuration encoding. Thus, the differential SF sensitivity between near-preferring and far-preferring clusters supported a primary difference between cortical mechanisms specialized for near versus far space, rather than a secondary selectivity based on “LVF versus UVF” representations.

### Experiment 4: SF preference in near- versus far-preferring clusters in 3-D stimuli

We next tested whether the higher sensitivity to 2-D low SFs in near-preferring compared with far-preferring clusters (as predicted by our hypothesis, and as supported by the results of Experiment 3) also generalized to 3-D cues. Stimuli were 3-D checkerboards generated using RDS, in which 3-D check size varied across the experimental blocks (i.e., 3 × 4 vs 6 × 8 vs 12 × 16 vs 24 × 32; see Materials and Methods). It was not possible to directly convert and compare 3-D configuration to 1-D spatial frequency (Experiment 3). Nevertheless, fundamental spatial frequencies in the 3 × 4, 6 × 8, 12 × 16, and 24 × 32 stimulus configurations include 0.075, 0.15, 0.3, and 0.6 c/deg (i.e., relatively low SF), along either horizontal or vertical directions. This means that, compared with Experiment 3 (in which we found a difference in response to low-SF, but not high-SF, stimuli in near- vs far-preferring clusters), Experiment 4 had a more limited range of stimuli. Therefore, rather than a significant cluster type × check size interaction (as we found in Experiment 3 as a significant cluster type × SF; see above), we expected a significant effect of cluster type. fMRI activity was measured relative to the 1 × 1 (zero-disparity) configuration.

**Figure 8.**
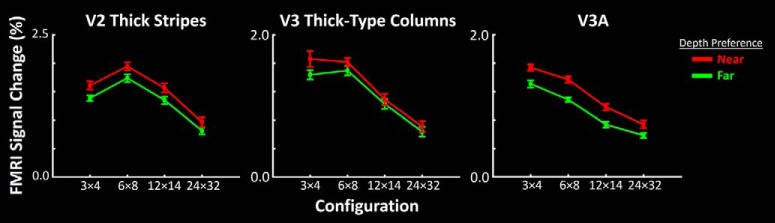
Stronger selectivity to lower SFs in 2-D stimuli in near-preferring compared with far-preferring clusters also generalizes to 3-D checks that are generated based on RDS. In V2, V3, and V3A, near-preferring clusters (red) show a stronger selectivity for larger 3-D checks, compared with far-preferring clusters (green). As in Figure 6, area V3A here showed a preference for lower SFs compared with areas V2 and V3. Error bars represent 1 SEM.

Figure 8 shows the responses evoked by these 3-D checkerboards in near- and far-preferring clusters in V3A, along with responses in earlier visual areas V2 and V3. In all three areas, stereoselective columns showed a stronger response to larger 3-D check configurations (i.e., 3 × 4 and 6 × 8) compared with the smaller ones (i.e., 12 × 16 and 24 × 32). Moreover, in all three areas, near-preferring clusters showed more sensitivity to the 3-D stimulus configuration, compared with far-preferring clusters. Furthermore, we noticed an apparent shift in the preferred stimulus configuration, from a 6 × 8 to a 3 × 4 configuration, between V2 to V3 to V3A, as we found previously based on 2-D gratings (see Experiment 3). A three-factor repeated-measures ANOVA (check size, cluster type, and area) showed significant effects of check size (*F*_(3,18)_ = 27.83, *p* < 10^−5^), cluster type (*F*_(1,6)_ = 12.80, *p* = 0.01), and area × check size (*F*_(6,36)_ = 16.68, *p* < 10^−3^; Table 4). Thus, the differences in SF sensitivity between the near- and far-preferring clusters were not limited to their responses to 2-D stimuli; the differences also extended to more complicated 3-D cues, despite the broad stimulus differences between 3-D checks and 2-D gratings.

**Table 4.** Check size (3-D structures) sensitivity across near- versus far-preferring clusters within areas V2, V3, and V3A (Experiment 4)

	*F* Value	*p* Value
Area (V2 vs V3 vs V3A)	5.30	0.03
Cluster type (near- vs far-preferring stereoselective cluster)	12.81	0.01
Check size (3 × 4 vs 6 × 8 vs 12 × 16 vs 24 × 32)	27.87	<10^−5^
Area × cluster type	0.64	0.49
Area × check size	16.68	<10^−3^
Cluster type × check size	1.26	0.31
Area × cluster type × check size	0.75	0.49

**Figure 9.**
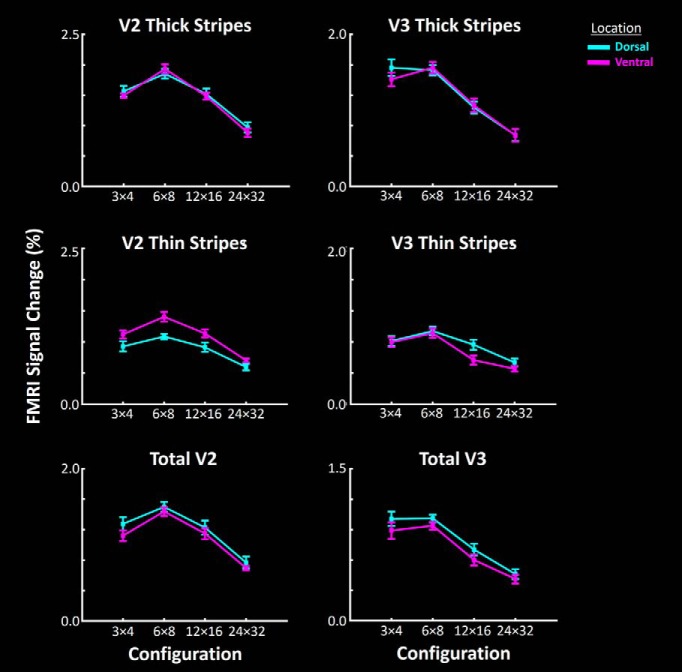
Response to RDS-based 3-D stimuli, with various check sizes measured within dorsal versus ventral regions of areas V2 and V3. In contrast to the difference between near- and far-preferring clusters (Fig. 8), we did not find any significant difference between activity evoked within dorsal versus ventral regions, regardless of their depth selectivity. This result suggests that the stronger response to larger check sizes within near-preferring compared with far-preferring clusters (Experiment 4) is not a second-order phenomenon arising from a potential difference between activity evoked within dorsal versus ventral regions. Other details are similar to those in Figure 7.

As in Experiment 3, we also checked whether a difference in check size sensitivity was found between LVF and UVF stereoselective regions, regardless of their near versus far depth preference (Fig. 9). Again, a three-factor repeated-measures ANOVA [check size, area, and cluster type (dorsal vs ventral)] confirmed significant effects of check size (*F*_(4,18)_ = 20.19, *p* < 10^−4^), and area × check size (*F*_(3,18)_ = 14.30, *p* < 10^−3^), without a significant effect of cluster type (*F*_(1,6)_ = 0.08, *p* = 0.79) and/or interaction between this effect and the effect of other independent factors (*F* < 1.26, *p* > 0.32; Table 5).

**Table 5.** Check size (3-D structures) sensitivity across dorsal versus ventral stereoselective columns of areas V2 and V3 (Experiment 4)

	*F* Value	*p* Value
Area (V2 vs V3)	10.40	0.02
Cluster type (dorsal vs ventral stereoselective clusters)	0.08	0.79
Check size (3 × 4 vs 6 × 8 vs 12 × 16 vs 24 × 32)	20.19	<10^−5^
Area × cluster type	<0.01	0.98
Area × check size	14.30	<10^−4^
Cluster type × check size	1.26	0.31
Area × cluster type × check size	0.73	0.46

Similar results were found when we applied this analysis to activity within dorsal versus ventral color-selective columns, and also the cortical representation of LVF and UVF, regardless of their stereoselectivity/color selectivity (Table 6). Other than a stronger check size sensitivity in ventral compared with dorsal color-selective columns (i.e., opposite to the pattern expected from the vertical asymmetry in configuration encoding), we did not find any difference between activity evoked across the ROIs. Thus, these results support our hypothesis that sensitivity to check size (3-D configuration) differs between near- and far-preferring clusters; it was not a secondary effect of sampling from the cortical representations of UVF versus LVF (see Discussion).

**Table 6.** Check size (3-D structures) sensitivity across dorsal versus ventral portions of areas V2 and V3 (Experiment 4)

	Color-selective columns		Whole visual area	
	*F* Value	*p* Value	*F* Value	*p* Value
Area (V2 vs V3)	47.21	<10^−3^	16.92	<0.01
Cluster type (dorsal vs ventral)	0.55	0.49	1.16	0.32
Check size (3 × 4 vs 6 × 8 vs 12 × 16 vs 24 × 32)	10.51	<0.01	13.67	<0.01
Area × cluster type	5.69	0.05	<0.01	0.96
Area × check size	8.05	<0.01	13.71	<10^−3^
Cluster type × check size	1.44	0.28	0.56	0.59
Area × cluster type × check size	3.65	0.05	0.70	0.49

## Discussion

The statistics of natural scenes, and a typical observer's extensive experience viewing them, are thought to shape visual perception. Although psychophysical studies have provided much evidence for such a bias (Previc, 1990; Yang and Purves, 2003), related physiological evidence is rare, often limited to activity measurements across large-scale brain regions using conventional fMRI techniques (Furmanski and Engel, 2000; Nasr and Tootell, 2012a). Here, we instead measured fMRI activity at a finer spatial scale (i.e., cortical clusters and columns) and demonstrated an association between (1) the level of vertical asymmetry in global perception (a phenomenon linked to higher relevance of low-SF visual feature in the LVF compared with the UVF) and (2) fMRI activity evoked within V3A near- and far-preferring clusters. This analysis at a smaller scale revealed cortical selectivity that would not have been visible at the larger spatial scale of conventional fMRI. Thus, our results highlight the insights available from studying fine-scale cortical structures in understanding the impact of natural scene statistics in shaping human behavior.

### Functional organization of the visual system is shaped by statistics of natural scenes

The visual system processes many different stimuli, more or less concurrently. However, the relevance of different visual stimuli for survival can vary significantly. For instance, objects that are physically nearer to the observer are often more relevant for survival (in terms of both threat and food acquisition), compared with objects that are located farther away. Such “near” stimuli appear more frequently within the LVF compared with the UVF (Yang and Purves, 2003). Consistent with this, previous studies in humans (Nasr and Tootell, 2018) and nonhuman primates (Adams and Zeki, 2001; Tanabe et al., 2005; Chen et al., 2008) have shown that clusters of near-preferring neurons are more frequently distributed within those cortical regions that represent the LVF, compared with those that represent the UVF. This systematic variation in the distribution of depth-sensitive neural clusters may enable the visual system to more effectively allocate its limited neural resources across the visual field (Previc, 1990; Yang and Purves, 2003).

The current results extend those previous findings by demonstrating a differential visual processing within near- and far-preferring clusters. Specifically, it has been proposed that visual blurring increases the relevance of low-SF components in visual perception, especially at near rather than far distances (Previc, 1990). Our findings of differential low-SF preference between near- and far-preferring clusters support the hypothesis that the human visual system may have “adapted” to the statistics of natural scenes by enhancing low-SF processing within near-preferring compared with far-preferring clusters. Our findings are also consistent with the interpretation that humans perceive near stimuli based more on spatially graded borders, compared with sharp borders (Marshall et al., 1996; Mather and Smith, 2000; Sprague et al., 2016).

Moreover, here we showed that the stronger low-SF response in near-preferring clusters, compared with far-preferring clusters, is not limited to 1-D gratings (Experiment 3). Rather, the bias extended to response to 3-D shapes, which were more naturalistic than the 1-D gratings. This generalization was evident despite the differences among (1) the spatial configuration, (2) the size of variation in stimuli used in Experiment 3 versus 4, and (3) the impact of these differences on the statistical analysis. Specifically, in Experiment 3, the 1-D stimuli covered a wider SF range compared with the 3-D stimulus used in Experiment 4. The results of Experiment 3 showed a significant interaction between the effects of SF × cluster type, suggestive of a stronger effect of cluster type on low compared with higher SFs (Fig. 6). In Experiment 4, we found only a significant effect of cluster type (rather than shape × cluster type interaction; Fig. 8), likely due to the narrower range of variations in stimulus shape in this test compared with Experiment 3. Thus, the results of both experiments suggest a stronger preference for low SFs in near-preferring compared with far-preferring stimuli.

### Near versus far or dorsal versus ventral?

Here we report a stronger selectivity within near-preferring compared with far-preferring clusters in V3A at low SFs. However, in lower-tier areas (e.g., areas V2 and V3), near-preferring clusters are more frequently distributed within dorsal visual areas compared with ventral visual areas (Nasr and Tootell, 2018). Therefore, it could be argued that this selectivity difference between near- and far-preferring clusters is a second-order phenomenon compared with low-SF selectivity differences between dorsal and ventral cortical regions.

To address this, we tested for a differential low-SF selectivity of dorsal versus ventral disparity-selective clusters, in all experiments. None of these tests showed a significant difference between activity evoked within dorsal versus ventral disparity-selective clusters. Moreover, we found a higher low SF-selective response within near-preferring compared with far-preferring clusters of area V3A, in which there is no systematic dorsal–ventral difference in the distribution of near- and far-preferring clusters. Thus, it is highly unlikely that our main findings arise secondarily from a difference between dorsal and ventral regions.

### Why V3A?

Sensitivity to horizontal binocular disparity is one important cue for visual depth (i.e., visual distance from the observer). Disparity-sensitive (stereoselective) activity has been reported in multiple visual areas, including V2, V3, V3A, and V4, in electrophysiological and imaging studies in nonhuman primates (Adams and Zeki, 2001; Hinkle and Connor, 2001; Thomas et al., 2002; Watanabe et al., 2002; Tsao et al., 2003; Chen et al., 2008; Anzai et al., 2011; Li et al., 2019) and in neuroimaging studies in humans (Tsao et al., 2003; Neri et al., 2004; Goncalves et al., 2015; Nasr et al., 2016; Nasr and Tootell, 2018). In a previous study, by taking advantage of high-field, high-resolution neuroimaging techniques, we found that clusters of such disparity-selective neurons show a strong selectivity for low-SF visual components (Tootell and Nasr, 2017). Interestingly, among the early retinotopic visual areas in humans, V3A showed the highest concentration of disparity-selective columns (Tootell and Nasr, 2017) and is highly active in a broad range of disparity tests (Tsao et al., 2003; Neri et al., 2004; Bridge and Parker, 2007; Cottereau et al., 2011; Goncalves et al., 2015). Furthermore, V3A showed a stronger low SF-selective response compared with lower visual areas V2 and V3 (Tootell and Nasr, 2017).

The current results extend our previous findings in human V3A by showing a significant correlation between the disparity-selective response within V3A (but not lower-stage areas) and the level of vertical asymmetry in global processing (measured behaviorally). These findings are consistent with previous studies that suggested a close link between activity evoked in human V3A (compared with the earlier visual areas) and the subjects' depth perception (Backus et al., 2001).

### Selective attention versus sensory processing

Multiple psychophysical studies have suggested that the stronger global processing in the LVF (compared with the UVF) is accompanied by relatively stronger attentional modulation in those regions that represent the LVF compared with the UVF (de Gonzaga Gawryszewski et al., 1987; Previc, 1990; Thomas and Elias, 2011). Thus, one might also expect a stronger attentional modulation of activity in near-preferring compared with far-preferring neural clusters.

Our “sensory” hypothesis tested here (i.e., differential low-SF selectivity within near- vs far-preferring clusters) and the alternative hypothesis of “attention modulation” (also within near- vs far-preferring clusters) are not mutually exclusive. However, it is unlikely that our findings are solely due to differential attention modulation, for several reasons. Specifically, in our neuroimaging tests, the impact of attention was reduced by requiring our subjects to perform a “dummy” attention task, detecting a change in either color or shape in a small fixation target, regardless of the much larger stimuli on screen. Also, our stimuli were either 2-D, presented within the frontoparallel plane relative to the fixation spot (Experiment 3), or 3-D, spanning near and far distances over an equivalent range (Experiment 4). Therefore, such simple geometrical stimuli are not expected to evoke differential attentional modulation within near- versus far-preferring clusters. Furthermore, we did not find any significant difference between the activity evoked within those regions that represented the LVF versus those that represented the UVF, in either of our tests. Thus, it is unlikely that our findings solely reflected a differential impact of attentional modulation, in either near- versus far-preferring clusters or LVF versus UVF.

Nevertheless, it remains possible that the difference in low-SF selectivity between near- and far-preferring cluster is enhanced when a subject's attention is directed toward global processing. Similar effects have been reported at macroscopic (area/region) spatial scales within face- and scene-selective regions, during face and scene discrimination tasks (O'Craven et al., 1999; Nasr and Tootell, 2012b). Further studies are required to clarify the effect of attention on the vertical asymmetry in global perception.

### Limitations

Here we focused on a specific aspect of global–local processing, the interaction between the perception of upper and lower visual fields and spatial frequency. It is widely recognized that multiple additional visual cues can affect global and local processing, including holistic processing (Tanaka and Farah, 2003; Young et al., 2013) and/or motion coherence (Williams and Sekuler, 1984; Siegel and Andersen, 1988). However, it is not yet known whether (or which of) these other cues show biases that are consistent with environmental constraints, as suggested here. Importantly, other biases in visual sensitivity also have been linked to environmental biases in natural image statistics (Furmanski and Engel, 2000; Sasaki et al., 2006; Rajimehr et al., 2011; Nasr and Tootell, 2012a; Nasr et al., 2014). This supports the general hypothesis that the statistics of natural scenes help to shape neural processing within human visual cortex.

## References

[B1] Adams DL, Zeki S (2001) Functional organization of macaque V3 for stereoscopic depth. J Neurophysiol 86:2195–2203. 10.1152/jn.2001.86.5.2195 11698511

[B2] Anzai A, Chowdhury SA, DeAngelis GC (2011) Coding of stereoscopic depth information in visual areas V3 and V3A. J Neurosci 31:10270–10282. 10.1523/JNEUROSCI.5956-10.2011 21753004 PMC3143190

[B3] Backus BT, Fleet DJ, Parker AJ, Heeger DJ (2001) Human cortical activity correlates with stereoscopic depth perception. J Neurophysiol 86:2054–2068. 10.1152/jn.2001.86.4.2054 11600661

[B4] Boynton GM, Engel SA, Glover GH, Heeger DJ (1996) Linear systems analysis of functional magnetic resonance imaging in human V1. J Neurosci 16:4207–4221. 10.1523/JNEUROSCI.16-13-04207.1996 8753882 PMC6579007

[B5] Brainard DH (1997) The psychophysics toolbox. Spat Vis 10:433–436. 10.1163/156856897X00357 9176952

[B6] Bridge H, Parker AJ (2007) Topographical representation of binocular depth in the human visual cortex using fMRI. J Vis 7(14):15, 1–14. 10.1167/7.14.15 18217810

[B7] Chen G, Lu HD, Roe AW (2008) A map for horizontal disparity in monkey V2. Neuron 58:442–450. 10.1016/j.neuron.2008.02.032 18466753 PMC2441920

[B8] Christman SD (1993) Local-global processing in the upper versus lower visual fields. Bull Psychon Soc 31:275–278. 10.3758/BF03334927

[B9] Cottereau BR, McKee SP, Ales JM, Norcia AM (2011) Disparity-tuned population responses from human visual cortex. J Neurosci 31:954–965. 10.1523/JNEUROSCI.3795-10.2011 21248120 PMC3298090

[B10] Courtney SM, Ungerleider LG, Keil K, Haxby JV (1997) Transient and sustained activity in a distributed neural system for human working memory. Nature 386:608–611. 10.1038/386608a0 9121584

[B11] Dale AM, Buckner RL (1997) Selective averaging of rapidly presented individual trials using fMRI. Hum Brain Mapp 5:329–340. 10.1002/(SICI)1097-0193(1997)5:5<329::AID-HBM1>3.0.CO;2-5 20408237

[B12] Dale AM, Fischl B, Sereno MI (1999) Cortical surface-based analysis. I. Segmentation and surface reconstruction. Neuroimage 9:179–194. 10.1006/nimg.1998.0395 9931268

[B13] de Gonzaga Gawryszewski L, Riggio L, Rizzolatti G, Umiltá C (1987) Movements of attention in the three spatial dimensions and the meaning of “neutral” cues. Neuropsychologia 25:19–29. 10.1016/0028-3932(87)90040-6 3574647

[B14] Dumoulin SO, Wandell BA (2008) Population receptive field estimates in human visual cortex. Neuroimage 39:647–660. 10.1016/j.neuroimage.2007.09.034 17977024 PMC3073038

[B15] Engel SA, Glover GH, Wandell BA (1997) Retinotopic organization in human visual cortex and the spatial precision of functional MRI. Cereb Cortex 7:181–192. 10.1093/cercor/7.2.181 9087826

[B16] Fischl B (2012) FreeSurfer. Neuroimage 62:774–781. 10.1016/j.neuroimage.2012.01.021 22248573 PMC3685476

[B17] Fischl B, Sereno MI, Dale AM (1999) Cortical surface-based analysis. II: inflation, flattening, and a surface-based coordinate system. Neuroimage 9:195–207. 10.1006/nimg.1998.0396 9931269

[B18] Fischl B, Salat DH, Busa E, Albert M, Dieterich M, Haselgrove C, van der Kouwe A, Killiany R, Kennedy D, Klaveness S, Montillo A, Makris N, Rosen B, Dale AM (2002) Whole brain segmentation: automated labeling of neuroanatomical structures in the human brain. Neuron 33:341–355. 10.1016/S0896-6273(02)00569-X 11832223

[B19] Flevaris AV, Bentin S, Robertson LC (2010) Local or global? attentional selection of spatial frequencies binds shapes to hierarchical levels. Psychol Sci 21:424–431. 10.1177/0956797609359909 20424080 PMC2861790

[B20] Friston KJ, Holmes AP, Price CJ, Büchel C, Worsley KJ (1999) Multisubject fMRI studies and conjunction analyses. Neuroimage 10:385–396. 10.1006/nimg.1999.0484 10493897

[B21] Furmanski CS, Engel SA (2000) An oblique effect in human primary visual cortex. Nat Neurosci 3:535–536. 10.1038/75702 10816307

[B22] Gaska JP, Jacobson LD, Pollen DA (1988) Spatial and temporal frequency selectivity of neurons in visual cortical area V3A of the macaque monkey. Vision Res 28:1179–1191. 10.1016/0042-6989(88)90035-1 3253990

[B23] Gattass R, Sousa AP, Rosa MG (1987) Visual topography of V1 in the cebus monkey. J Comp Neurol 259:529–548. 10.1002/cne.902590404 3597827

[B24] Goncalves NR, Ban H, Sánchez-Panchuelo RM, Francis ST, Schluppeck D, Welchman AE (2015) 7 tesla FMRI reveals systematic functional organization for binocular disparity in dorsal visual cortex. J Neurosci 35:3056–3072. 10.1523/JNEUROSCI.3047-14.2015 25698743 PMC4331627

[B25] Greve DN, Fischl B (2009) Accurate and robust brain image alignment using boundary-based registration. Neuroimage 48:63–72. 10.1016/j.neuroimage.2009.06.060 19573611 PMC2733527

[B26] Hinkle DA, Connor CE (2001) Disparity tuning in macaque area V4. Neuroreport 12:365–369. 10.1097/00001756-200102120-00036 11209951

[B27] Hubel DH, Wiesel TN (1974) Uniformity of monkey striate cortex: a parallel relationship between field size, scatter, and magnification factor. J Comp Neurol 158:295–305. 10.1002/cne.901580305 4436457

[B28] Julesz B (1971) Foundations of cyclopean perception. Chicago: University of Chicago.

[B29] Keil B, Triantafyllou C, Hamm M, Wald LL (2010) Design optimization of a 32-channel head coil at 7T. In: ISMRM-ESMRMB Joint Annual Meeting 2010: Stockholm, Sweden, 1–7 May 2010/monograph, p 1943 Red Hook, NY: Curran Associates.

[B30] LaGasse LL (1993) Effects of good form and spatial frequency on global precedence. Percept Psychophys 53:89–105. 10.3758/BF03211718 8433909

[B31] Levine MW, McAnany JJ (2005) The relative capabilities of the upper and lower visual hemifields. Vision Res 45:2820–2830. 10.1016/j.visres.2005.04.001 16051308

[B32] Li X, Zhu Q, Janssens T, Arsenault JT, Vanduffel W (2019) In vivo identification of thick, thin, and pale stripes of macaque area V2 using submillimeter resolution (f)MRI at 3 T. Cereb Cortex 29:544–560. 10.1093/cercor/bhx337 29300915

[B33] Liu T, Heeger DJ, Carrasco M (2006) Neural correlates of the visual vertical meridian asymmetry. J Vis 6:1294–1306. 10.1167/6.11.12 17209736 PMC1864963

[B34] Marshall JA, Burbeck CA, Ariely D, Rolland JP, Martin KE (1996) Occlusion edge blur: a cue to relative visual depth. J Opt Soc Am A Opt Image Sci Vis 13:681–688. 10.1364/josaa.13.000681 8867752

[B35] Mather G, Smith DR (2000) Depth cue integration: stereopsis and image blur. Vision Res 40:3501–3506. 10.1016/S0042-6989(00)00178-4 11115677

[B36] Minini L, Parker AJ, Bridge H (2010) Neural modulation by binocular disparity greatest in human dorsal visual stream. J Neurophysiol 104:169–178. 10.1152/jn.00790.2009 20445027 PMC2904223

[B37] Nasr S, Tootell RB (2012a) A cardinal orientation bias in scene-selective visual cortex. J Neurosci 32:14921–14926. 10.1523/JNEUROSCI.2036-12.2012 23100415 PMC3495613

[B38] Nasr S, Tootell RB (2012b) Role of fusiform and anterior temporal cortical areas in facial recognition. Neuroimage 63:1743–1753. 10.1016/j.neuroimage.2012.08.031 23034518 PMC3472036

[B39] Nasr S, Tootell RBH (2018) Visual field biases for near and far stimuli in disparity selective columns in human visual cortex. Neuroimage 168:358–365. 10.1016/j.neuroimage.2016.09.012 27622398 PMC5346058

[B40] Nasr S, Liu N, Devaney KJ, Yue X, Rajimehr R, Ungerleider LG, Tootell RB (2011) Scene-selective cortical regions in human and nonhuman primates. J Neurosci 31:13771–13785. 10.1523/JNEUROSCI.2792-11.2011 21957240 PMC3489186

[B41] Nasr S, Echavarria CE, Tootell RB (2014) Thinking outside the box: rectilinear shapes selectively activate scene-selective cortex. J Neurosci 34:6721–6735. 10.1523/JNEUROSCI.4802-13.2014 24828628 PMC4019792

[B42] Nasr S, Polimeni JR, Tootell RB (2016) Interdigitated color- and disparity-selective columns within human visual cortical areas V2 and V3. J Neurosci 36:1841–1857. 10.1523/JNEUROSCI.3518-15.2016 26865609 PMC4748071

[B43] Neri P, Bridge H, Heeger DJ (2004) Stereoscopic processing of absolute and relative disparity in human visual cortex. J Neurophysiol 92:1880–1891. 10.1152/jn.01042.2003 15331652

[B44] Niebauer CL, Christman SD (1998) Upper and lower visual field differences in categorical and coordinate judgments. Psychon Bull Rev 5:147–151. 10.3758/BF03209471

[B45] O'Craven KM, Downing PE, Kanwisher N (1999) fMRI evidence for objects as the Us of attentional selection. Nature 401:584–587. 10.1038/44134 10524624

[B46] Pelli DG (1997) The VideoToolbox software for visual psychophysics: transforming numbers into movies. Spat Vis 10:437–442. 10.1163/156856897X00366 9176953

[B47] Polimeni JR, Fischl B, Greve DN, Wald LL (2010) Laminar analysis of 7T BOLD using an imposed spatial activation pattern in human V1. Neuroimage 52:1334–1346. 10.1016/j.neuroimage.2010.05.005 20460157 PMC3130346

[B48] Polimeni JR, Bhat H, Witzel T, Benner T, Feiweier T, Inati SJ, Renvall V, Heberlein K, Wald LL (2016) Reducing sensitivity losses due to respiration and motion in accelerated echo planar imaging by reordering the autocalibration data acquisition. Magn Reson Med 75:665–679. 10.1002/mrm.25628 25809559 PMC4580494

[B49] Previc FH (1990) Functional specialization in the lower and upper visual fields in humans: its ecological origins and neurophysiological implications. Behav Brain Sci 13:519–575. 10.1017/S0140525X00080018

[B50] Rajimehr R, Devaney KJ, Bilenko NY, Young JC, Tootell RB (2011) The “parahippocampal place area” responds preferentially to high spatial frequencies in humans and monkeys. PLoS Biol 9:e1000608. 10.1371/journal.pbio.1000608 21483719 PMC3071373

[B51] Robertson LC, Egly R, Lamb MR, Kerth L (1993) Spatial attention and cuing to global and local levels of hierarchical structure. J Exp Psychol Hum Percept Perform 19:471–487. 10.1037//0096-1523.19.3.471 8331311

[B52] Sasaki Y, Rajimehr R, Kim BW, Ekstrom LB, Vanduffel W, Tootell RB (2006) The radial bias: a different slant on visual orientation sensitivity in human and nonhuman primates. Neuron 51:661–670. 10.1016/j.neuron.2006.07.021 16950163

[B53] Sereno MI, Dale AM, Reppas JB, Kwong KK, Belliveau JW, Brady TJ, Rosen BR, Tootell RB (1995) Borders of multiple visual areas in humans revealed by functional magnetic resonance imaging. Science 268:889–893. 10.1126/science.7754376 7754376

[B54] Sereno MI, Lutti A, Weiskopf N, Dick F (2013) Mapping the human cortical surface by combining quantitative T(1) with retinotopy. Cereb Cortex 23:2261–2268. 10.1093/cercor/bhs213 22826609 PMC3729202

[B55] Shulman GL, Wilson J (1987) Spatial frequency and selective attention to local and global information. Perception 16:89–101. 10.1068/p160089 3671045

[B56] Shulman GL, Sullivan MA, Gish K, Sakoda WJ (1986) The role of spatial-frequency channels in the perception of local and global structure. Perception 15:259–273. 10.1068/p150259 3797200

[B57] Siegel RM, Andersen RA (1988) Perception of three-dimensional structure from motion in monkey and man. Nature 331:259–261. 10.1038/331259a0 3336437

[B58] Skrandies W (1987) The upper and lower visual field of man: electrophysiological and functional differences. In: Progress in sensory physiology, Vol 8 (Autrum H, Ottoson D, Perl ER, Schmidt RF, Shimazu H, Willis WD, eds). Berlin, Heidelberg: Springer-Verlag 10.1007/978-3-642-71060-5_1

[B59] Smith AT, Singh KD, Williams AL, Greenlee MW (2001) Estimating receptive field size from fMRI data in human striate and extrastriate visual cortex. Cereb Cortex 11:1182–1190. 10.1093/cercor/11.12.1182 11709489

[B60] Sprague WW, Cooper EA, Reissier S, Yellapragada B, Banks MS (2016) The natural statistics of blur. J Vis 16(10):23, 1–27. 10.1167/16.10.23 27580043 PMC5015925

[B61] Tanabe S, Doi T, Umeda K, Fujita I (2005) Disparity-tuning characteristics of neuronal responses to dynamic random-dot stereograms in macaque visual area V4. J Neurophysiol 94:2683–2699. 10.1152/jn.00319.2005 16000525

[B62] Tanaka JW, Farah MJ (2003) The holistic representation of faces. In: Perception of faces, objects, and scenes: analytic and holistic processes (Peterson MA, Rhodes G, eds), pp 53–74. Westport, CT: Praeger 10.1093/acprof:oso/9780195313659.003.0003

[B63] Thomas NA, Elias LJ (2011) Upper and lower visual field differences in perceptual asymmetries. Brain Res 1387:108–115. 10.1016/j.brainres.2011.02.063 21362412

[B64] Thomas OM, Cumming BG, Parker AJ (2002) A specialization for relative disparity in V2. Nat Neurosci 5:472–478. 10.1038/nn837 11967544

[B65] Tootell RBH, Nasr S (2017) Columnar segregation of magnocellular and parvocellular streams in human extrastriate cortex. J Neurosci 37:8014–8032. 10.1523/JNEUROSCI.0690-17.2017 28724749 PMC5559769

[B66] Tsao DY, Vanduffel W, Sasaki Y, Fize D, Knutsen TA, Mandeville JB, Wald LL, Dale AM, Rosen BR, Van Essen DC, Livingstone MS, Orban GA, Tootell RB (2003) Stereopsis activates V3A and caudal intraparietal areas in macaques and humans. Neuron 39:555–568. 10.1016/S0896-6273(03)00459-8 12895427

[B67] Wade AR, Brewer AA, Rieger JW, Wandell BA (2002) Functional measurements of human ventral occipital cortex: retinotopy and colour. Philos Trans R Soc Lond B Biol Sci 357:963–973. 10.1098/rstb.2002.1108 12217168 PMC1693014

[B68] Watanabe M, Tanaka H, Uka T, Fujita I (2002) Disparity-selective neurons in area V4 of macaque monkeys. J Neurophysiol 87:1960–1973. 10.1152/jn.00780.2000 11929915

[B69] Williams DW, Sekuler R (1984) Coherent global motion percepts from stochastic local motions. Vision Res 24:55–62. 10.1016/0042-6989(84)90144-5 6695508

[B70] Yacoub E, Harel N, Ugurbil K (2008) High-field fMRI unveils orientation columns in humans. Proc Natl Acad Sci U S A 105:10607–10612. 10.1073/pnas.0804110105 18641121 PMC2492463

[B71] Yang Z, Purves D (2003) A statistical explanation of visual space. Nat Neurosci 6:632–640. 10.1038/nn1059 12754512

[B72] Young AW, Hellawell D, Hay DC (2013) Configurational information in face perception. Perception 42:1166–1178. 10.1068/p160747n 24601030

